# Why does health literacy matter, and for whom? Explaining the differentiating impact of health literacy on vaccine attitudes

**DOI:** 10.3389/fpsyg.2025.1470654

**Published:** 2025-03-27

**Authors:** Maruša Lubej, Andrej Kirbiš

**Affiliations:** Department of Sociology, Faculty of Arts, University of Maribor, Maribor, Slovenia

**Keywords:** health literacy, vaccine hesitancy, vaccine attitudes, vaccine myths, socioeconomic, sociodemographic

## Abstract

**Introduction:**

Vaccination has substantially reduced the spread and severity of infectious diseases. Despite its efficacy, vaccine hesitancy remains a global challenge, often linked to inadequate health literacy and unfavorable vaccine attitudes. Understanding the mechanisms through which health literacy influences vaccine-related attitudes is crucial because it could inform policy interventions aimed at fostering more favorable vaccine attitudes.

**Method:**

The present cross-sectional study of Slovenian adults (*n* = 3,360) examined the impact of health literacy on vaccine attitudes, focusing on the mediating role of beliefs in vaccine myths and the moderating effects of gender, education, economic status, healthcare training, and self-rated health.

**Results:**

Using mediation and moderated mediation models, we found that health literacy positively influences vaccine attitudes both directly and indirectly by reducing beliefs in myths. The relationship between health literacy and vaccine attitudes is moderated by healthcare training, with stronger positive effect among individuals with such training. Additionally, the negative effect of health literacy on beliefs in myths is stronger among individuals with higher education.

**Discussion:**

Our findings indicate that broader educational inequalities should be addressed to ensure that the positive effect of health literacy on vaccine attitudes is distributed more equitably across groups with different educational levels and fields of expertise.

## 1 Introduction

Vaccination is one of the most effective public health interventions, significantly reducing the prevalence and impact of infectious diseases (Horney, 2023). However, despite its proven efficacy, vaccine hesitancy, defined as delay and refusal of vaccinations, remains a barrier to immunization success worldwide (Dubé et al., 2013; Lane et al., 2018; Fadda et al., 2020; Caserotti et al., 2021; Pourrazavi et al., 2023).

Health literacy, the ability to obtain, understand, and use information to make appropriate health decisions (Nutbeam, 2000, p. 263), is a key factor influencing health behaviors, including vaccination (Nutbeam, 2000; Sun et al., 2013; Aaby et al., 2017). Several studies show that low health literacy is linked to higher vaccine hesitancy (Berkman et al., 2011; Magon et al., 2021; Zhang et al., 2022; Fenta et al., 2023; Çetin and Sögüt, 2024) and negative attitudes toward vaccines (Aslan et al., 2023; Fenta et al., 2023). While vaccine attitudes capture general perceptions of vaccines’ importance, safety, and effectiveness (Jovanović and Lazić, 2023), beliefs in myths about vaccination focus on beliefs in specific false statements about vaccines (Omisakin et al., 2023). However, in a systematic review, Lorini et al. (2018) concluded that the link between health literacy and vaccination remains unclear.

Prior research indicates that higher levels of health literacy are linked to lower vaccine hesitancy and more positive vaccine attitudes (Berkman et al., 2011; Magon et al., 2021; Zhang et al., 2022; Fenta et al., 2023). Additionally, the Health Literacy Survey 2019–2021 (HLS19) found that higher vaccination health literacy—defined as “individuals’ knowledge, motivation, and skills to find, understand, and evaluate immunization-related information in order to make adequate immunization decisions” (Griebler et al., 2021, p. 314)—is associated with better risk knowledge across all seven countries analyzed. Additionally, in six of these countries, it is linked to greater vaccine confidence and improved disease risk perception (Griebler et al., 2021). However, Kricorian et al. (2022) found that those who perceived the COVID-19 vaccine as unsafe not only expressed vaccine hesitancy but also reported difficulties understanding scientific information. Moreover, these individuals were more likely to believe vaccine myths and less likely to use trustworthy scientific information sources. Thus, Kricorian et al. (2022) suggest that negative COVID-19 vaccine attitudes may partly be due to lower health literacy in underprivileged communities. Additionally, health literacy might have a differentiated effect on vaccine attitudes among different groups (Lorini et al., 2018; Zhang et al., 2022).

Therefore, in the present study, we examine whether beliefs in vaccine myths mediate the relationship between health literacy and vaccine attitudes. Second, we explore whether the potential moderators (gender, education, healthcare training, economic status, educational level, and health status) modify the impact of health literacy on beliefs in myths and vaccine attitudes.

### 1.1 Explaining the link between health literacy and vaccine attitudes

Health literacy increases vaccination rates (Guclu et al., 2019; Kitur et al., 2022; Galvin et al., 2023, pp. 27–45) and decreases vaccine hesitancy (Magon et al., 2021; Zhang et al., 2022; Yang et al., 2023). One of the characteristics of vaccine-hesitant individuals are their false beliefs about vaccines. Research indicates a negative association between believing in vaccine myths and vaccine attitudes (Kricorian et al., 2022; Nah et al., 2024). For example, Kricorian et al. (2022) found that individuals who perceive vaccines as unsafe are more likely to believe in myths about vaccines (Kricorian et al., 2022). Myths about vaccines, such as the belief that vaccines overload the immune system, or cause diseases, can undermine public confidence in vaccines and reduce vaccination rates (Dubé et al., 2015; Wilson et al., 2015).

Health literacy may play an important role in vaccination outcomes through various pathways. Among other, it may reduce beliefs in myths about vaccines, e.g., being unsafe and inefficient (Gray et al., 2005; Ishikawa and Yano, 2008; Cheng and Nishikawa, 2024). Higher health literacy can equip individuals with the skills to critically evaluate health information and discern credible sources (Gray et al., 2005; Ishikawa and Yano, 2008). As a result, individuals with higher health literacy are less likely to believe in vaccine myths and misinformation (Carletto et al., 2024) more likely to have positive vaccine attitudes and are less vaccine-hesitant (Berkman et al., 2011; Fenta et al., 2023). Based on the existing evidence we propose that beliefs in vaccine myths play a mediating role in the relationship between vaccine literacy and favorable vaccine attitudes.

*Hypothesis 1*: Higher health literacy is associated with more positive vaccine attitudes through reduced beliefs in vaccine myths.

### 1.2 Moderators of health literacy’s impact on vaccine myth beliefs and vaccine attitudes

Besides the need to explain how health literacy impacts health outcomes and behavior, including vaccine attitudes, scholars have recently also focused on for whom health literacy matters, i.e., on potentially differentiated impact of health literacy on vaccine-related attitudes and behavior among different social groups. In their systematic review, Lorini et al. (2018) suggest that several factors, including age, country, and type of vaccine may affect the link between health literacy and vaccine hesitancy. For example, White et al. (2008) found that low literacy decreases health-preventive behavior such as regular health check-ups and adherence to preventive measures for adults aged 65 and older, but not for younger adults. Zhang et al. (2022) reported that increasing health literacy reduces vaccine hesitancy in China, though this effect disappears under high stress.

Based on these findings, scholars have called for further studies on the role of social and economic factors as potential moderators of the impact of health literacy on health outcomes (Geboers et al., 2016). To design effective public health policies, including those aiming to increase health literacy and improving vaccination rates, it is critical to examine whether health literacy’s role in health outcomes differs depending on the social or economic characteristics of the groups individuals are members of.

Specifically, several potential demographic and economic characteristics might play a role in determining the relevance of health literacy. For example, demographic and socioeconomic factors, such as gender, educational level, and economic status may moderate the impact of health literacy on beliefs in myths. Gender differences in information processing and health-seeking behavior may lead to the stronger negative effect of health literacy on beliefs in myths and a stronger positive effect on vaccine attitudes among females, since women tend to be more engaged, involved, attentive, and better informed when making health-related decisions (Courtenay, 2000; Ek, 2015). In addition, women have greater trust in doctors than men (Wiltshire et al., 2011; Gopichandran and Sakthivel, 2021), which increases vaccine uptake (Soveri et al., 2020; Viskupič et al., 2022). We therefore propose that among females, the beneficial effect of health literacy on beliefs in vaccine myths and on vaccine attitudes is increased.

*Hypothesis 2:* The negative effect of health literacy on beliefs in vaccine myths is stronger among females (H2a), and the positive effect of health literacy on vaccine attitudes is stronger among females (H2b).

Socioeconomic resources may also modify the role of health literacy. Education, for example, provides skills that make it easier to find and interpret reliable information to promote one’s health and make informed decisions (Adler and Newman, 2002), thereby potentially enhancing the effect of health literacy on not believing in vaccine myths and expressing favorable vaccine attitudes. Similarly, sufficient financial resources enable access to quality health information and services (Devaux, 2015; Utomo et al., 2023), which may further strengthen the negative effects of health literacy on beliefs in vaccine myths, and the positive effects of health literacy on vaccine acceptance. Taken together, we expect that education and economic status moderate the effect of health literacy on beliefs in vaccine myths and on vaccine attitudes.

*Hypothesis 3:* The negative effect of health literacy on beliefs in vaccine myths is stronger among individuals with higher educational levels (H3a), and the positive effect of health literacy on vaccine attitudes is stronger among individuals with higher education levels (H3b).

*Hypothesis 4:* The negative effect of health literacy on belief in vaccine myths is stronger among individuals with fewer financial difficulties (H4a), and the positive effect of health literacy on vaccine attitudes is stronger among individuals with fewer financial difficulties (H4b).

Similar to a higher education level, whether someone has trained in a healthcare profession may also play a significant moderating role in affecting the link between health literacy, belief in myths, and vaccine attitudes. Health literacy has been examined among the general public and various health professions, such as doctors (Todorovic et al., 2019; Stock et al., 2021; Mohamed-Yassin et al., 2023), nurses (Gu et al., 2022), and other clinical practitioners (Shiferaw and Mehari, 2019; Elyamani and Hammoud, 2020). Research has found that health literacy is higher among individuals who have healthcare training or are employed in healthcare, in comparison with the general population (Hofer-Fischanger et al., 2020). Moreover, Panayotova et al. (2023) found that vaccines are widely accepted amongst healthcare workers worldwide and that most healthcare professionals recommend vaccination to their patients. Verulava and Verulava (2024) suggest that a higher vaccine acceptance rate among healthcare workers is influenced by their professional knowledge and training, which enhances their vaccine literacy and perception of risk, leading to greater willingness to get vaccinated. Because training or working in healthcare contributes to increased health literacy and better vaccine knowledge (Gu et al., 2022; Verulava and Verulava, 2024), we expect that healthcare educational background reduces susceptibility to vaccine myths and enhances the link between high health literacy and positive vaccine attitudes.

*Hypothesis 5:* The negative effect of health literacy on belief in vaccine myths is stronger among people who have trained in a healthcare profession (H5a). Furthermore, the positive effect of health literacy on vaccine attitudes is stronger among individuals who have trained in a healthcare profession (H5b).

Lastly, health is another critical factor that might moderate the relationship between health literacy and vaccine outcomes. The most often cited reason for the general population’s support for vaccination is healthcare professionals’ advice (Haverkate et al., 2012; Yaqub et al., 2014). People with poor health tend to use healthcare services more often than healthier people (Barreto et al., 2006; Berra et al., 2006). Therefore, people with poor health may be directly influenced by healthcare providers’ advice and information (including about vaccines) more often than healthier people, which might strengthen the negative effect of health literacy on beliefs in myths, and the positive effect of health literacy on vaccine attitudes among the less healthy.

*Hypothesis 6:* The negative effect of health literacy on beliefs in vaccine myths is stronger among individuals with poorer health (H6a), and the positive effect of health literacy on vaccine attitudes is stronger among individuals with poorer health (H6b).

### 1.3 Study aim

Existing studies have investigated the relationships between health literacy and vaccine attitudes (Berkman et al., 2011; Magon et al., 2021; Zhang et al., 2022; Fenta et al., 2023), beliefs in myths and vaccine attitudes (Kricorian et al., 2022; Nah et al., 2024), and health literacy and belief in myths (Gray et al., 2005; Ishikawa and Yano, 2008; Kricorian et al., 2022; Cheng and Nishikawa, 2024). However, to our knowledge, no research has examined whether beliefs in vaccine myths mediates the relationship between health literacy and vaccine attitudes, and whether this mediation effect varies across different social and economic groups. Considering the gaps in the literature on why and for whom health literacy has an impact on vaccine attitudes, this study aimed to examine (1) whether beliefs in vaccine myths are a mechanism linking health literacy and vaccine attitudes. In addition, we analyzed (2) to what extent the beneficial role of health literacy differs among social and economic groups. Thus, we tested the moderated mediation model of the impact of health literacy on vaccine attitudes through beliefs in myths, with gender, education, economic status, healthcare training, and health status as moderators.

## 2 Methodology

### 2.1 Participants and procedure

Our dataset was obtained from the Slovenian Institute of Public Health (Vrdelja et al., 2022), which collaborated, as a representative of Slovenia, in the cross-country study HLS 19 (HLS19 Consortium of the WHO Action Network M-POHL, 2021). The sample included Slovenians, aged 18 years and older. The data was collected with the probability sampling method, using a two-stage stratified sampling from the Central Population Register. Data collection started in March 2020 and ended in August 2020. Participants were initially invited to complete an online questionnaire, with options for computer-assisted personal interviewing or paper-based responses for those who did not participate online. Out of the 6,000 individuals selected, contact was established with 5,585, resulting in a 56% response rate with 3,360 completed questionnaires. The sample was weighted by gender, age, statistical region, and education level.

### 2.2 Measures

#### 2.2.1 Health literacy

We assessed health literacy using an index with 16 items from the HLS19-Q16 questionnaire (HLS19 Consortium of the WHO Action Network M-POHL, 2021; Vrdelja et al., 2022), which consists of three factors: health care/treatment, disease prevention and health promotion/encouragement (Mialhe et al., 2022; Pedro et al., 2023). Participants responded on a four-point scale (1 = very difficult; 4 = very easy). All items of the questionnaire are shown in Table 1. The items were averaged to create a composite health literacy score.

**Table 1:** Items used to assess health literacy.

Item
… to find information on treatments of illnesses that concern you?
… to find out where to get professional help when you are ill?
… to understand what a doctor says to you?
… to understand your doctor’s or pharmacist’s instruction on how to take a prescribed medicine?
… to judge if you may need to get a second opinion from another doctor?
… to use information your doctor gives to you to make decisions about your illness?
… to act on advice from your doctor or pharmacist?
… to find information on how to handle mental health problems?
… to understand information about unhealthy habits such as smoking, low physical activity or drinking too much alcohol?
… to understand information about recommended health screenings or examinations?
… to judge if the information on health risks in the mass media is reliable?
… to decide how you can protect yourself from illness using information from the mass media?
… to find information about activities that are good for your mental health and well-being?
… to understand advice concerning your health from family or friends?
… to understand information in the mass media on how to improve your health?
… to judge which everyday habits affect your health?

Source: Vrdelja et al. (2022). Items were rated from 1 (very difficult) to 4 (very easy).

#### 2.2.2 Beliefs in myths

Beliefs in myths about vaccinations were assessed using an index of three items: “Vaccines overload and weaken the immune system,” “Vaccines can cause the diseases against which they protect,” and “Vaccines often produce serious side effects (other than the normal and temporary reactions in the first few days)” (HLS19 Consortium of the WHO Action Network M-POHL, 2021; Vrdelja et al., 2022). Participants indicated whether those statements are true (1) or false (2). We recoded and averaged those items to create an index for beliefs in myths, ranging from 0 (all correct answers) to 1 (no correct answers), with a higher score indicating belief in more myths.

#### 2.2.3 Vaccine attitudes

Vaccine attitudes were measured using three items on a 4-point Likert scale (1 = strongly agree; 4 = strongly disagree). These items included the following statements: “Vaccinations are important to protect myself and my children,” “Overall I think vaccinations are effective,” and “Overall I think vaccinations are safe” (HLS19 Consortium of the WHO Action Network M-POHL, 2021; Vrdelja et al., 2022). The scale was reversed so that a lower score indicates less favorable attitudes towards vaccines. The items were then averaged to create a composite vaccine attitudes score.

#### 2.2.4 Moderators

Five moderators were included in the model: gender (0 = male, 1 = female), education was recoded into four categories (1 = elementary or less, 2 = secondary, 3 = tertiary (BA or similar), 4 = tertiary (MA, PhD, specialization)), respondents’ ability to pay bills was recoded into two categories (0 = very easy and easy, 1 = hard and very hard), self-rated health was recoded into three categories (1 = very poor and poor health, 2 = neither good nor poor health, 3 = good and very good health) and healthcare training was assessed by asking whether respondents had ever been trained to work in healthcare (0 = no, 1 = yes).

#### 2.2.5 Statistical analyses

Following the approach by Cameron et al. (2023), the missing values for any item were replaced with the mean of the other item ratings within each index. The responses were then averaged across all items in each index to generate a composite score for each participant. Consistent with Jakobsen et al. (2017) and Cameron et al. (2023), we chose not to conduct imputations for missing values due to the minimal percentage of missing data in our variables of interest (see Table 2). To assess the internal consistency of our three indices, we conducted a principal components analysis (PCA) and calculated Cronbach’s alpha. Before conducting PCA, we used Bartlett’s Test of Sphericity to confirm that factor analysis was appropriate and the Kaiser-Meyer-Olkin (KMO) Measure of Sampling Adequacy to assess the suitability of data for PCA (Williams et al., 2010).

**Table 2:** Descriptive statistics of the examined variables.

Variable	*N*	Min	Max	Mean	SD	Kurtosis	Category	%
Health literacy	3,360	1	4	3.10	0.43	1.11		
Beliefs in myths	3,360	0	1	0.32	0.38	−0.94		
Vaccine attitudes	3,294	1	4	3.11	0.61	0.67		
Gender (0 = male)	3,360	0	1			−1.98	Female	53.7%
							Male	46.3%
Paying Bills (0 = easy)	3,334	0	1			−1.87	Easy	58.9%
							Difficult	41.1%
Self-rated Health (2 = neither good nor poor health)	3,357	1	3			0.50	Good	66.7%
							Neither	26.4%
							Poor	6.9%
Education Level (4 = MA, PhD, specialization)	3,360	1	4			−0.09	Primary	14.5%
							Secondary	54.6%
							Tertiary (BA or similar)	27.3%
							Tertiary (MA, PhD, specialization)	3.7%
Trained to Work in Healthcare (0 = yes)	3,357	0	1			6.32	Yes	8.9%
							No	91.1%

Notes. Reference categories are indicated in parentheses within variable names. The table presents unweighted statistics.

Following Tadros et al. (2022) and Liu et al. (2024), we applied effect coding (−0.5, +0.5) to all moderator variables and we mean-centered the health literacy index to mitigate multicollinearity (Zhang et al., 2017). To further address this issue, we merged primary and secondary education into a single category. After these transformations, all VIF values fell below the commonly accepted threshold for severe multicollinearity (VIF < 10; Olsen et al., 2020).

**Figure 1:**
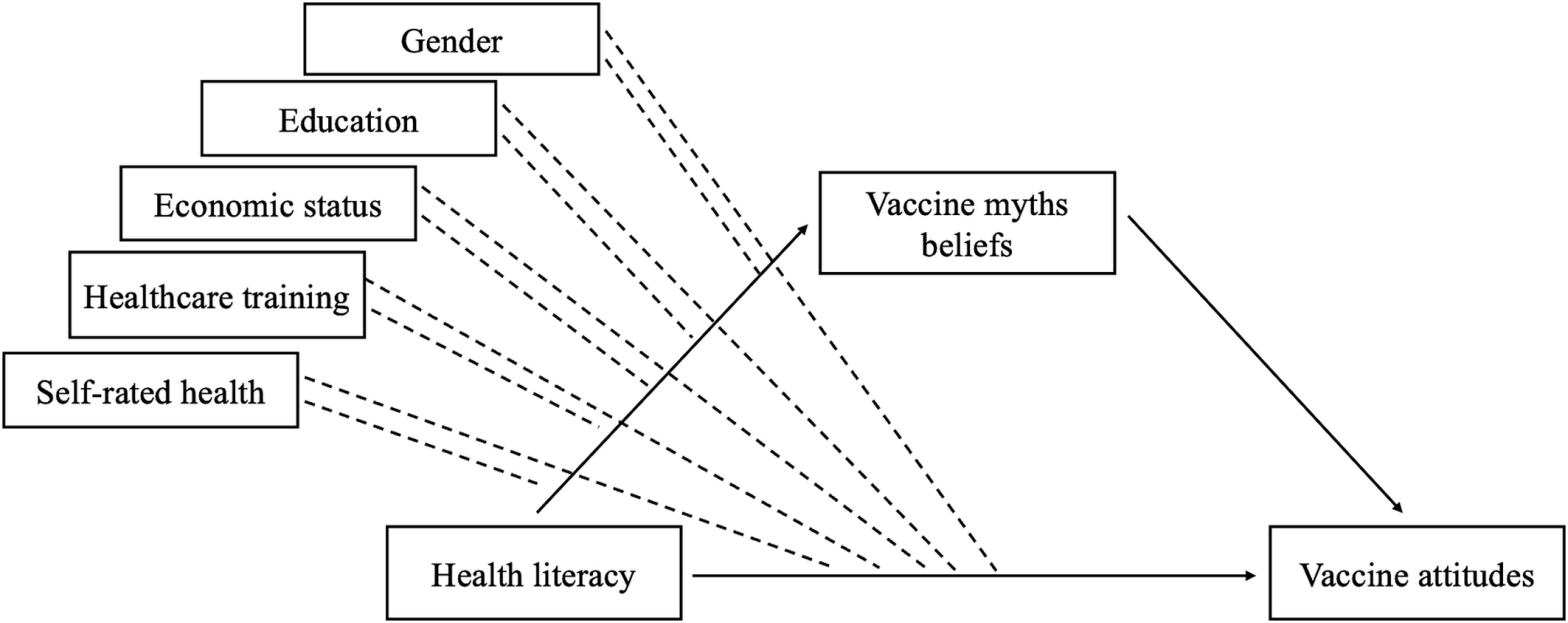
Hypothesized model of moderated mediation relationships.

To initially examine the relationships among study variables, we conducted Spearman’s rank correlation (*ρ*) for ordinal variables, point-biserial correlations (rpb) when one variable was binary, and phi coefficients (*φ*) when both variables were binary (see Table 3) (Pallant, 2020). We used RStudio (version 2024.04.0 + 735) and lavaan package to test the moderated and mediated relationships specified in the proposed model (see Figure 1). We first conducted preliminary analyses testing mediation without the moderators. Then, we tested our model with the moderators, using dummy variables for education (1 = elementary or less, 2 = secondary, 3 = tertiary (BA or similar), 4 = tertiary (MA, PhD, specialization) and self-rated health (1 = very poor and poor health, 2 = neither good nor poor health, 3 = good and very good health)) (we set tertiary education (MA, PhD, specialization) and group that rated their health as neither good nor bad as reference categories, see Table 2).

**Table 3:** Correlations among examined variables and PCA statistics for the indices.

**Variable**	**1.**	**2.**	**3.**	**4.**	**5.**	**6.**	**7.**	**8.**
1.Health Literacy	1.000							
2.Vaccine Attitudes	0.261 ***	1.000						
3.Beliefs in myths	−0.079 ***	−0.444 ***	1.000					
4.Paying Bills	−0.238 ***	−0.151 ***	0.123 ***	1.000				
5.Gender	0.064 ***	0.002	0.057 ***	0.055 ***	1.000			
6.Self-rated Health	0.261 ***	0.086 ***	−0.036 *	−0.275 ***	−0.046 **	1.000		
7.Education	0.273 ***	0.160 ***	−0.073 ***	−0.309 ***	0.005	0.270 ***	1.000	
8.Healthcare training	0.183 ***	0.079 ***	−0.032	−0.131 ***	0.137 ***	0.056 ***	0.131 ***	1.000
PCA KMO	0.950	0.743	0.684					
PCA factor loadings	0.54–0.73	0.89–0.93	0.79–0.82					
PCA eigenvalue	7.19	2.50	1.97					
PCA % Variance	45%	83%	66%					
Cronbach’s α	0.91	0.90	0.74					
Average r	0.41	0.75	0.48					
Bartlett’s test χ^2^ (df)	23631.35 (120) ***	6349.676 (3) ***	2179.57 (3) ***					

All correlations are Spearman’s rho coefficients (ρ), except when one variable is binary, in which case point-biserial correlations (r_pb_) are used, and when both variables are binary, in which case a phi coefficient (φ) is used. KMO=Kaiser-Meyer-Olkin Measure of Sampling Adequacy. Average *r*, average inter-item correlation. The table presents unweighted statistics. ***p < 0.001; **p < 0.01; *p < 0.05.

Following Peña and Slate (2006) and Kirshenbaum et al. (2023), we used gvlma to check for potential linear regression assumptions. Results indicated violations of normality in all regressions and nonlinearity in the relationship between health literacy and beliefs in myths. To address this nonlinearity, we included a quadratic term for health literacy in that path (Becker et al., 2019). After the inclusion of the quadratic term, the Link Function test indicated that the linearity assumption was satisfied. To mitigate other assumption deviations, we used Robust Maximum Likelihood Estimation (MLR), following (Robles et al., 2022). The MLR method provides robust standard errors and a scaled test statistic that are robust to non-normality and heteroscedasticity. The models were evaluated using the standardized root mean square residual (SRMR), comparative fit index (CFI), and root mean square error of approximation (RMSEA). A good model fit is indicated by CFI values greater than 0.90, while SRMR and RMSEA values of 0.07 or lower suggest a good fit (Carvalho et al., 2018). Unstandardized estimates were reported.

## 3 Results

### 3.1 Sample demographics and personal characteristics

Table 2 summarizes the sample demographics and personal characteristics.

The Health Literacy Index had a mean score of 3.10 (SD = 0.43), ranging from 1 to 4, indicating generally high health literacy among the participants. The Belief in Myths Index had a mean score of 0.32 (SD = 0.38), with scores ranging from 0 to 1, indicating a lower tendency to believe in myths. The Vaccine Attitudes Index had a mean score of 3.11 (SD = 0.61), indicating generally positive attitudes towards vaccines in our sample.

The sample consisted of 53.7% females and 46.3% males. The majority of participants reported ease in paying bills (58.4%), while 41.1% reported difficulty. In terms of self-rated health, 66.6% of the participants rated their health as (very) good, 26.3% as neither good nor poor, and 6.9% as (very) poor. Most participants had secondary education (54.6%), followed by tertiary education (BA or similar) (27.3%), primary education (14.5%), and MA, PhD, or specialization (3.7%). Additionally, 8.9% of the participants trained in a healthcare profession, while the remaining 91% were not.

### 3.2 Bivariate analysis and PCA

Table 3 presents correlations among the variables, along with statistics from principal component analyses (PCA) for the relevant measures. The Kaiser-Meyer-Olkin (KMO) Measure of Sampling Adequacy statistics indicated acceptable inter-correlation for structure detection, with KMO values of 0.950, 0.743, and 0.684 for the three components. All measures showed significant Bartlett’s tests of sphericity, χ2 > 2179.57, *p* < 0.0001. Each analysis extracted one principal component with item coefficients ranging from 0.54 to 0.93. Cronbach’s *α* values ranged from 0.74 to 0.91, indicating good internal consistency.

The correlations reveal significant relationships among the analyzed variables. Higher health literacy is associated with more favorable vaccine attitudes (*ρ* = 0.261, *p* < 0.001). Additionally, higher health literacy is positively associated with female gender (ρ = 0.064, *p* < 0.001), better self-rated health (ρ = 0.261, *p* < 0.001), higher education levels (ρ = 0.273, *p* < 0.001) and having received healthcare training (*r*_pb_ = 0.183, *p* < 0.001). Conversely, higher health literacy is linked to lower beliefs in myths about vaccinations (ρ = −0.079, *p* < 0.001) and less difficulty in paying bills (*r*_pb_ = −0.238, *p* < 0.001).

More favorable vaccine attitudes are associated with lower beliefs in vaccine myths (ρ = −0.444, *p* < 0.001). Additionally, more favorable vaccine attitudes are also associated with higher education levels (ρ = 0.160, *p* < 0.001), better self-rated health (ρ = 0.086, *p* < 0.001) and having received healthcare training (*r*_pb_ = 0.079, *p* < 0.001). Moreover, those with favorable vaccine attitudes have less difficulty in paying bills (*r*_pb_ = −0.151, *p* < 0.001).

Greater beliefs in myths about vaccinations are associated with more difficulty in paying bills (*r*_pb_ = 0.123, *p* < 0.001). Beliefs in myths are also higher among females (ρ = 0.057, *p* < 0.001) and those with lower education levels (ρ = −0.073, *p* < 0.001). Additionally, those with higher beliefs in myths tend to have worse self-rated health (ρ = −0.036, *p* < 0.05).

### 3.3 The mediation model

The results of mediation analysis are presented in Table 4. The mediation model demonstrated good fit (CFI = 1.000; RMSEA = 0.000; SRMR = 0.002).

**Table 4:** Mediation model: the impact of health literacy on belief in myths and vaccine attitudes.

Path	Estimate	*p*-value	95% CI [LL, UL]
Regressions			
Beliefs in myths ~ Health Literacy (a1)	−0.063	<0.001	[−0.094, −0.032]
Beliefs in myths ~ Health Literacy^2^ (a2)	−0.060	0.009	[−0.104, −0.015]
Vaccine Attitudes ~ Beliefs in myths (b)	−0.738	<0.001	[−0.798, −0.677]
Vaccine Attitudes ~ Health Literacy (c)	0.267	<0.001	[0.221, 0.314]
Defined parameters			
Indirect Effect (Linear: a1 * b)	0.047	<0.001	[0.023, 0.070]
Indirect Effect (Quadratic: a2 * b)	0.044	0.009	[0.011, 0.077]
Total Indirect Effect (a1 * b + a2 * b)	0.090	<0.001	[0.049, 0.132]
Total Effect (c + Total Indirect Effect)	0.358	<0.001	[0.294, 0.422]

Unstandardized estimates were reported. CFI = 1.000; RMSEA = 0.000; SRMR = 0.002. The table presents weighted statistics.

Health literacy was negatively associated with beliefs in myths through both a linear effect (a1: *β* = −0.063, *p* < 0.001, CI [−0.094, −0.032]) and a quadratic effect (a2: β = −0.060, *p* = 0.009, CI [−0.104, −0.015]). The negative quadratic term suggests a diminishing return effect in the relationship between health literacy and beliefs in myths, where the decline in beliefs in myths is less pronounced at higher levels of health literacy. Beliefs in myths, in turn, were significantly negatively associated with vaccine attitudes (b: *β* = −0.738, *p* < 0.001, CI [−0.798, −0.677]).

The total indirect effect of health literacy on vaccine attitudes via beliefs in myths (a1 * b + a2 * b) was significant (*β* = 0.090, *p* < 0.001, CI [0.049, 0.132]), with the positive linear (a1 * b) (*β* = 0.047, *p* < 0.001, CI [0.023, 0.070]) and non-linear (a2 * b) (*β* = 0.044, *p* = 0.009, CI [0.011, 0.077]) indirect effect. These findings suggest that both the linear and nonlinear components of health literacy play a role in shaping vaccine attitudes through their influence on beliefs in myths. The total effect of health literacy on vaccine attitudes, combining the direct and total indirect effects (c + Total Indirect Effect), was significant (*β* = 0.358, *p* < 0.001, CI [0.294, 0.422]). The percentage of the total effect that the total indirect effect accounts for is approximately 25.28%. These results support Hypothesis 1, as beliefs in vaccine myths significantly mediated the relationship between health literacy and vaccine attitudes.

### 3.4 The moderated mediation model

Next, the moderated mediation model was specified to examine whether the indirect effect of health literacy on vaccine attitudes through beliefs in myths is moderated by gender, education, economic status, healthcare training, health status. Model fit was good, as indicated by the fit indices (CFI = 1.000; RMSEA = 0.000; SRMR = 0.000). The results are displayed in Table 5.

**Table 5:** Moderated mediation model: the moderating effects of demographic and health-related factors.

Path	Estimate	*p*-value	95% CI [LL, UL]
Regressions			
Beliefs in myths ~ Health Literacy (a1)	−0.064	0.051	[−0.128, 0.000]
Beliefs in myths ~ Health Literacy^2^ (a2)	−0.029	0.266	[−0.081, 0.022]
Beliefs in myths ~ Health Literacy * Gender (i1)	0.037	0.270	[−0.029, 0.103]
Beliefs in myths ~ Health Literacy * Paying Bills (i2)	0.005	0.882	[−0.067, 0.078]
Beliefs in myths ~ Health Literacy * Health (poor) (i3)	0.024	0.675	[−0.089, 0.138]
Beliefs in myths ~ Health Literacy * Health (good) (i4)	−0.029	0.496	[−0.111, 0.054]
Beliefs in myths ~ Health Literacy * Education (pri., sec.) (i5)	0.179	0.014	[0.036, 0.323]
Beliefs in myths ~ Health Literacy * Education (BA) (i6)	0.146	0.049	[0.000, 0.292]
Beliefs in myths ~ Health Literacy * Health Training (i7)	−0.031	0.508	[−0.121, 0.060]
Vaccine Attitudes ~ Beliefs in myths (b1)	−0.740	<0.001	[−0.801, −0.679]
Vaccine Attitudes ~ Health Literacy (c1)	0.326	<0.001	[0.237, 0.415]
Vaccine Attitudes ~ Health Literacy * Gender (i8)	0.066	0.174	[−0.029, 0.162]
Vaccine Attitudes ~ Health Literacy * Paying Bills (i9)	−0.027	0.637	[−0.139, 0.085]
Vaccine Attitudes ~ Health Literacy * Health (poor) (i10)	−0.010	0.879	[−0.144, 0.123]
Vaccine Attitudes ~ Health Literacy * Health (good) (i11)	−0.018	0.764	[−0.137, 0.101]
Vaccine Attitudes ~ Health Literacy * Education (pri., sec.) (i12)	−0.063	0.538	[−0.262, 0.137]
Vaccine Attitudes ~ Health Literacy * Education (BA) (i13)	−0.042	0.692	[−0.249, 0.165]
Vaccine Attitudes ~ Health Literacy * Health Training (i14)	0.164	0.030	[0.016, 0.311]
Defined parameters			
Indirect Effect (Linear Contribution: a1 * b1)	0.047	0.051	[0.000, 0.095]
Indirect Effect (Quadratic Contribution: a2 * b1)	0.022	0.266	[−0.017, 0.060]
Total Indirect Effect (a1 * b1 + a2 * b1)	0.069	0.035	[0.005, 0.134]
Total Effect (c1 + Total Indirect Effect)	0.395	<0.001	[0.277, 0.513]

Unstandardized estimates were reported. CFI = 1.000; RMSEA = 0.000; SRMR = 0.000. The table presents weighted statistics.

The results show a marginally significant negative effect of health literacy on beliefs in myths (a1: *β* = −0.064, *p* = 0.051, CI [−0.128, 0.000]). However, the quadratic term for health literacy was non-significant in predicting beliefs in myths (a2: *β* = −0.029, *p* = 0.266, CI [−0.081, 0.022]). Additionally, similar to our model without the moderators, health literacy had a significant positive effect on vaccine attitudes (c1: *β* = 0.326, *p* < 0.001, CI [0.237, 0.415]) while beliefs in myths were significantly negatively associated with vaccine attitudes (b1: *β* = −0.740, *p* < 0.001, CI [−0.801, −0.679]).

The relationship between health literacy and beliefs in myths was significantly moderated by education. Specifically, a significant positive interaction between health literacy and primary/secondary education levels (i5: *β* = 0.179, *p* = 0.014, CI [0.036, 0.323]) and between health literacy and BA-level education (i6: *β* = 0.146, *p* = 0.049, CI [0.000, 0.292]) suggests that the negative relationship between health literacy and beliefs in myths is weaker for individuals with lower levels of education (primary/secondary and BA-level) compared to those with higher education (MA, PhD or specialization). This supports Hypothesis 3a, which posits that the negative effect of health literacy on beliefs in vaccine myths is stronger among those with higher educational levels.

Additionally, the path between health literacy and vaccine attitudes was significantly moderated by health training (i14: *β* = 0.164, *p* = 0.030, CI [0.016, 0.311]). This indicates that higher health literacy is more strongly associated with vaccine attitudes for people trained in a healthcare profession compared to those without such training, thus providing evidence for Hypothesis 5b.

Interactions involving gender, financial difficulty, and self-rated health were not significant (*p* > 0.05), rejecting H2, H4 and H6. These results suggest that, in this model, these moderators do not meaningfully influence the effect of health literacy on beliefs in myths and on vaccine attitudes.

While the linear indirect effect of health literacy on vaccine attitudes via beliefs in myths (a1 * b1) was significant (*β* = 0.047, p = 0.051, CI [0.000, 0.095]), the non-linear indirect effect (a2 * b) (*β* = 0.022, *p* = 0.266, CI [−0.017, 0.060]) was not. However, the total indirect effect was significant (*β* = 0.069, *p* = 0.035, CI [0.005, 0.134]) and so was the total effect (*β* = 0.395, *p* < 0.001, CI [0.277, 0.513]). After the moderators were added to the model, the percentage of the total effect that the total indirect effect accounts for is approximately 17.52%.

## 4 Discussion

Our study examined the role of health literacy in shaping vaccine attitudes, focusing on the mediating effect of belief in vaccine myths and the moderating roles of gender, education, economic status, healthcare training, and health status. We found that health literacy positively affects vaccine attitudes by reducing beliefs in vaccine myths. Additionally, this effect is moderated by education and healthcare training, while gender, economic status and self-rated health do not significantly moderate these relationships.

We showed that health literacy directly and indirectly, by reducing belief in myths, enhances positive vaccine attitudes. This aligns with previous studies showing that higher health literacy is linked to more favorable attitudes towards vaccines (Berkman et al., 2011; Magon et al., 2021; Zhang et al., 2022; Fenta et al., 2023). Moreover, our findings show that health literacy reduces belief in vaccine myths, which is consistent with previous research (Gray et al., 2005; Ishikawa and Yano, 2008; Kricorian et al., 2022; Cheng and Nishikawa, 2024). It may be easier for health literate individuals to distinguish credible sources from those that are not, which then makes them less prone to form beliefs in myths about vaccines (Ishikawa and Yano, 2008; Carletto et al., 2024). Furthermore, we found that belief in vaccine myths negatively impacts vaccine attitudes, which is also consistent with earlier findings (Kricorian et al., 2022; Nah et al., 2024). Individuals who believe in myths about vaccines may have negative perceptions of vaccine safety and efficacy, which can make people less likely to trust vaccine programs (Dubé et al., 2015; Wilson et al., 2015). By testing the mediation effect, we filled a research gap, showing how health literacy influences vaccine attitudes both directly and indirectly through belief in myths.

Besides analyzing why health literacy matters for vaccine attitudes, we also analyzed for whom it matters. Previous studies have shown that the impact of health literacy on health outcomes varies across different social groups (Geboers et al., 2016; Lorini et al., 2018; Zhang et al., 2022). We confirmed that in Slovenia, the effect of health literacy on vaccine attitudes is moderated by healthcare training. Specifically, the positive effect of health literacy on vaccine attitudes is stronger among people trained in a healthcare profession. Furthermore, we showed that the reduction in beliefs in myths due to higher health literacy is stronger for individuals with higher education compared to those with lower education levels. However, gender, economic status and self-rated health did not significantly moderate the effect of health literacy on beliefs in vaccine myths and vaccine attitudes.

Our findings have important policy implications. First, increasing health literacy may increase favorable vaccine attitudes, and, since the direct effect of health literacy becomes less negative as health literacy increases, interventions should prioritize low-literacy populations where gains are strongest. However, increasing health literacy across groups with varying education levels and professions may widen the gap in vaccine myths and attitudes among groups that differ in education levels, and those with and without healthcare education. To reduce the gap between those groups, policymakers should consider implementing nationwide interventions in elementary schools or curriculum changes, covering topics that build basic scientific understanding and trust in science from an early age. However, as indicated by Chang and Lauderdale (2009) such interventions might not be enough. Ultimately, initiatives aimed at ensuring access to high-quality education could lead to more equal effects of high health literacy across educational groups.

Although our study provides insight into the mechanisms of the relationship between health literacy, beliefs in vaccine myths and vaccine attitudes, and the differentiated effects of health literacy, several limitations of the study must be considered when interpreting the results. Our study is cross-sectional, which limits us from inferring causation. Second, while we included several moderators in our analysis, there may be other factors, such as age and type of vaccine (Lorini et al., 2018), or specific dimension of health, such as stress (Zhang et al., 2022), that may influence the relationships between health literacy, beliefs in myths, and vaccine attitudes. Future studies should therefore explore additional moderators (Lorini et al., 2018) and the role of specific types of health literacy (e.g., ehealth literacy; see Shiferaw and Mehari, 2019). Third, socioeconomic factors such as education and financial difficulties are oversimplified in the analyses and categorized in ways that may oversimplify their influence. For example, education levels may not fully capture access to quality education or critical thinking skills. Fourth, while the results from this study incorporate the non-linearity present between health literacy and beliefs in myths, they may not fully capture thresholds across all relationships. Similarly to Reumers et al. (2024), we acknowledge that threshold effects may exist. However, although we accounted for non-linearity to some extent, investigating potential non-linearity in depth was not a primary focus of this study and could be explored further using more complex multiple-group or nonlinear modeling approaches. Lastly, cross-cultural comparisons should be carried out to understand how cultural contexts may influence the relationship between health literacy and vaccine attitudes.

## 5 Conclusion

Our findings suggest that health literacy positively influences vaccine attitudes both directly and indirectly by reducing belief in vaccine myths. We found that the effect of health literacy on beliefs in myths is stronger among individuals with higher education. Additionally, the positive effect of health literacy on vaccine attitudes is stronger among people with healthcare training compared to those without such training. Our findings stress the need to address inequalities in education to ensure that the positive impact of health literacy on vaccine attitudes and other health-related outcomes does not exacerbate disparities among education levels and fields of expertise, particularly between healthcare and non-healthcare backgrounds. In future studies, other moderators (e.g., age) and the role of different health literacy types in influencing beliefs in vaccine myths and vaccine acceptance should be examined. Additionally, cross-cultural research should be implemented.

## Data Availability

The datasets presented in this article are not readily available due to legal project requirements established by NIJZ. Requests to access the datasets should be directed to the National Institute of Public Health Slovenia at: https://nijz.si/.
